# Comparative evaluation and architectural enhancement of a genetic algorithm-tuned fuzzy logic battery control in microgrid energy management

**DOI:** 10.1038/s41598-026-43620-7

**Published:** 2026-03-19

**Authors:** Meryem Meliani, Abdelhafid El Attafi, Abdellah El Barkany, Sofiane Kichou

**Affiliations:** 1 Czech Technical University in Prague, University Centre for Energy Efficient Buildings, 1024 Trinecka St., 27343 Bustehrad, Czech Republic; 2 LIMAS Laboratory, Faculty of Sciences Dhar El Mahraz, Sidi Mohammed Ben Abdellah University, 30003 Fez, Morocco; 3 Research Laboratory in Science and Engineering, Faculty of Science and Techniques, Sidi Mohammed Ben Abdellah University, B.P. 2202, Route d’Imouzzer, Fez, Morocco

**Keywords:** Microgrid, Genetic algorithm, Fuzzy logic tuning, Battery control, Energy management, Energy science and technology, Engineering

## Abstract

Efficient battery energy management remains a key challenge in renewable-integrated microgrids due to nonlinear battery dynamics, intermittent generation, and variable load demand. This paper investigates a fuzzy logic-based battery control strategy for a hybrid microgrid comprising photovoltaic generation, wind energy, battery energy storage, and a bidirectional grid interface. A two-phase control framework is adopted. In Phase 1, a genetic algorithm (GA) is used to tune an established fuzzy logic controller under identical operating conditions previously reported for particle swarm optimization (PSO) and backtracking search algorithm (BSA) approaches, enabling a fair comparative evaluation. In Phase 2, the controller architecture is enhanced by embedding battery charge-discharge and grid import-export decisions directly within the fuzzy inference system using real-time power imbalance and state-of-charge as inputs. Simulation results show that, while GA converges more slowly than BSA, all tuning methods achieve comparable battery current and state-of-charge regulation in Phase 1. In contrast, the enhanced control architecture in Phase 2 yields more coherent energy management behavior, improved utilization of renewable energy, reduced unnecessary battery cycling, and stable electrical performance. The results demonstrate that controller architecture has a greater impact on practical microgrid energy management performance than tuning strategy alone.

**Supplementary Information:**

The online version contains supplementary material available at 10.1038/s41598-026-43620-7.

## Introduction

### Background and motivation

The increasing penetration of renewable energy sources (RES) has accelerated the transition toward decentralized and intelligent power systems, positioning microgrids (MGs) as a key paradigm in modern power networks^1,2^. By integrating distributed renewable generation, energy storage systems, and grid interconnections, MGs offer greater flexibility and resilience than conventional centralized grids^3^. However, the stochastic nature of RES and time-varying load demand introduces significant challenges in maintaining power balance and system stability^4,5^.

Among MG components, battery energy storage systems play a crucial role in mitigating the intermittency of renewable energy sources and enhancing grid interaction^6,7^. Effective battery control must address nonlinear battery dynamics, operational state-of-charge (SOC) constraints, and uncertain operating conditions. Conventional control strategies, such as proportional-integral-derivative (PID) control, are simple to implement but exhibit limited robustness under nonlinear and time-varying conditions^8,9^. Advanced techniques such as model predictive control (MPC) and adaptive control can improve performance but often require accurate system models and impose high computational burdens, limiting their real-time applicability in practical MG deployments^5^.

Fuzzy logic control (FLC) has therefore gained significant attention for managing MG energy, owing to its ability to handle nonlinearities and uncertainties without requiring precise mathematical models^2,6,7^. Nevertheless, the effectiveness of FLC-based strategies strongly depends on the appropriate tuning of membership functions and rule bases, motivating the integration of intelligent optimization techniques.

### Literature review and research gaps

Energy management and battery control have been extensively studied in the context of MGs, given the increasing penetration of renewable energy sources and the critical role of energy storage systems. Early MG energy management strategies primarily relied on conventional control techniques, such as proportional-integral-derivative (PID) controllers^10^, owing to their simplicity and ease of implementation. However, several studies have reported that PID-based approaches exhibit limited robustness when applied to systems characterized by nonlinear dynamics, renewable intermittency, and rapid load variations^8,9^. As a result, their effectiveness in renewable-dominated MGs is often compromised.

To overcome the limitations of classical controllers, model-based control strategies such as model predictive control (MPC), adaptive control, and robust control have been proposed. MPC-based energy management strategies explicitly account for system constraints and future predictions, leading to improved performance in terms of power balancing and constraint handling^5^. Nevertheless, these approaches typically require accurate system models and involve significant computational effort, which can limit their applicability in real-time MG operation, particularly for low-cost or embedded controllers. Adaptive and robust control techniques have also been explored, but their performance often depends on the accuracy of parameter estimation and predefined uncertainty bounds^11^.

In recent years, intelligent control techniques have gained increasing attention, with fuzzy logic control (FLC) emerging as a promising solution for microgrid energy management. FLC offers a rule-based framework that handles system nonlinearities and uncertainties without requiring precise mathematical models. Meliani et al.^6^ applied fuzzy logic to regulate energy flows in MGs and demonstrated improved robustness compared to classical controllers. Similarly, Arcos-Aviles et al. in^7^ reviewed fuzzy-based energy management strategies for residential MGs and highlighted their effectiveness in smoothing grid power profiles. A comparative overview of classical, model-based, and intelligent control strategies commonly applied in microgrid energy management is summarized in Table 1, highlighting the trade-offs between modeling requirements, robustness, and real-time feasibility.

**Table 1:** Comparison of control strategies for microgrid energy management.

Control strategy	Model requirement	Handling of nonlinearity & uncertainty	Adaptability to RES variability	Real-time feasibility	Key limitations
PID Control	Requires linearized or simplified model	Poor	Low	High	Ineffective under nonlinear dynamics and high RES intermittency
Model Predictive Control (MPC)	Requires accurate predictive model	Good (model-based)	Moderate–High	Medium–Low (solver dependent)	High computational burden, sensitive to model mismatch
Adaptive Control	Nominal model with parameter estimation	Moderate	Moderate	Medium	Stability depends on adaptation law, sensitive to noise
Robust Control	Structured uncertainty bounds required	Good within defined limits	Low–Moderate	Medium	Conservative design, limited flexibility
Fuzzy Logic Control (FLC)	No explicit mathematical model	High	High	High	Performance depends on rule base and membership tuning

Despite these advantages, the performance of FLC-based controllers strongly depends on the proper design of membership functions (MFs) and rule bases. Manual tuning methods are often heuristic and lack scalability, motivating the integration of optimization algorithms to systematically tune fuzzy parameters. Among these, population-based metaheuristic algorithms have been widely adopted. Faisal et al. in^12^ proposed a fuzzy charging-discharging controller optimized using the backtracking search algorithm (BSA) and demonstrated improved battery SOC regulation in microgrid applications. In a subsequent study, the same authors employed particle swarm optimization (PSO) to tune fuzzy parameters, reporting improved convergence and power quality performance^3^.

Genetic algorithms (GA) have also been widely applied to fuzzy controller tuning due to their strong global search capability. Herrera et al*.*^13^ laid the foundation for GA-based fuzzy tuning, while more recent studies, such as^14^ and^15^, demonstrated the effectiveness of GA-optimized fuzzy controllers in renewable-integrated microgrids. Differential evolution (DE)^16^, ant colony optimization (ACO)^17,18^, artificial bee colony optimization^19^, whale optimization algorithms^20,21^, and grey wolf optimization techniques^22^ have further expanded the range of optimization tools available for fuzzy control design.

Additional studies have introduced advanced and hybrid optimization techniques to further improve convergence speed and control accuracy in microgrid energy management applications. These include hybrid swarm-based and evolutionary approaches for microgrid energy management and optimal power flow problems, enhanced metaheuristic algorithms for renewable-integrated systems, and multi-objective optimization frameworks addressing operational efficiency and power quality. Nayak et al.^23^ investigated power balance regulation and battery state-of-charge (SOC) control in renewable-based microgrids using a metaheuristic-tuned fuzzy controller, demonstrating effective SOC regulation and improved power balancing through simulation studies. In subsequent works^24,25^, the same authors extended this approach by applying different metaheuristic optimization techniques to tune FLC for grid-connected MGs with the primary objective of maintaining battery SOC within safe operating limits while managing power flow under variable load and generation conditions. Mishra et al.^26^ emphasized overall power coordination and operational stability among MG components using an intelligent, optimization-based control framework. Similarly, Prusty et al.^27^ proposed an optimization-based energy management strategy to enhance battery SOC regulation and power flow coordination in renewable-integrated microgrids. In both cases, simulation results confirmed improved operational stability and effective energy management under the tested scenarios.

Despite these promising results, the reported analyses remain largely limited to specific fuzzy controller architectures, with performance improvements primarily attributed to the selected optimization techniques. The influence of alternative tuning strategies and design choices for controller structure is not explicitly examined. A comparative summary of commonly used fuzzy tuning algorithms, including their main strengths and limitations, is provided in Table 2.

**Table 2:** Comparison of fuzzy logic controller tuning techniques for microgrid energy management.

Optimization method	Optimization type	Convergence characteristics	Suitability for FLC tuning	EMS-level strengths	Key limitations	Representative references
PSO	Population-based	Fast initial convergence	High	Simple implementation, widely used in MG EMS	Risk of premature convergence	^3,28–31^
BSA	Population-based	Moderate–Fast	High	Strong global exploration	Less intuitive parameter control	^12,32–34^
GA	Population-based	Moderate (exploration-oriented)	High	Robust for complex, nonlinear search spaces	Requires careful parameter tuning	^13–15,35,36^
DE	Population-based	Moderate	High	Efficient for continuous parameters	Sensitive to mutation strategy	^16,37–40^
ACO	Population-based	Moderate	Medium	Effective for discrete decision problems	Complex adaptation to continuous MFs	^17,18,41–44^
Hybrid / Enhanced Methods	Hybrid	Fast–Moderate	High	Improved convergence and robustness	Increased algorithmic complexity	^14,45,46^

From the above review, several gaps can be identified. Most existing studies focus on optimizing fuzzy controllers with fixed input–output structures tailored to specific MG configurations. The influence of fuzzy controller architectural design, such as the choice of input–output variables and decision logic, remains insufficiently explored. In addition, many FLC-based MG controllers regulate battery behavior using continuous control outputs, while grid import and export decisions are often handled externally or through simplified threshold logic. This separation can reduce coordination between battery operation and grid interaction in grid-connected MGs. Furthermore, despite the abundance of optimization techniques, direct comparative evaluations under identical system conditions are scarce, making it difficult to objectively assess the relative merits of tuning algorithms. These gaps motivate the present study, which investigates both the effect of the optimization strategy and the impact of architectural enhancements to fuzzy controllers on the practical performance of MG energy management.

### Aim and contributions

In this study, population-based metaheuristic algorithms are adopted to address the nonlinear, nonconvex, and simulation-based nature of FLC tuning, for which analytical gradients are unavailable. Since no optimization algorithm is universally optimal and algorithm suitability depends on problem characteristics, population-based methods are selected for their robust global exploration capability and reduced sensitivity to suboptimal solutions. Non-population-based optimization techniques are acknowledged but are generally more appropriate for smooth or convex problems.

This paper proposes a two-phase FL-based battery energy management strategy for a hybrid MG integrating PV generation, wind energy, battery energy storage, and a bidirectional grid interface. The specific contributions of this work are as follows:

- A fair and reproducible comparative evaluation of metaheuristic tuning methods is conducted by applying a GA to an established FL battery controller under identical system conditions previously reported for PSO and BSA-based tuning approaches^12^. This enables a direct comparison of convergence behavior and control performance without confounding effects from modeling differences.
- An enhanced fuzzy controller architecture is proposed, in which the control strategy is restructured from continuous current-based regulation to a unified, rule-based energy management scheme using real-time power imbalance (ΔP) and battery SOC as inputs, and discrete charging, discharging, grid import, and grid export decisions as outputs.
- The influence of controller architecture on MG energy management effectiveness is systematically analyzed, demonstrating that structural modifications to the fuzzy control framework can yield more significant performance improvements than tuning alone, particularly in terms of battery utilization efficiency and reduced dependence on grid power.
- Comprehensive simulation-based validation is provided, including convergence analysis, battery current, SOC evolution, and electrical waveform assessment at the grid interface, confirming the robustness and practical relevance of the proposed approach under dynamic operating conditions.

### Paper organization

The remainder of this paper is organized as follows. Section "Methodology" presents the MG configuration and the proposed FL-based control methodology, including the optimization framework. Section "Results" discusses simulation results and comparative performance analysis. Section "Discussions" provides an in-depth discussion of the findings and their implications. Finally, Section "Conclusion" concludes the paper and outlines directions for future research.

## Methodology

### System description and microgrid configuration

**Fig. 1:**
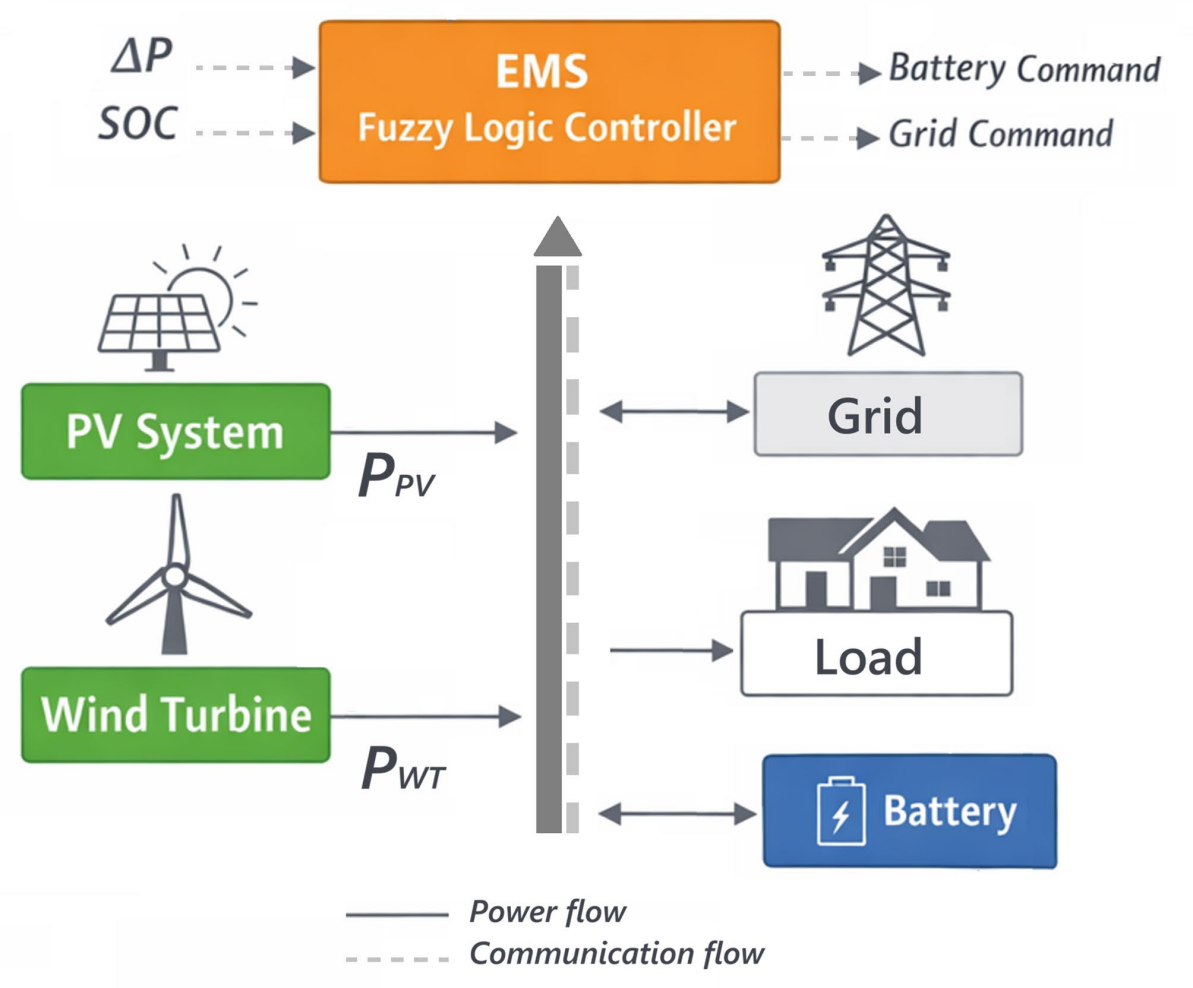
The structure of the MG system.

The MG system considered in this study is a hybrid renewable-based configuration comprising PV generation, wind energy conversion, a battery energy storage system (BESS), local loads, and a bidirectional connection to the main utility grid. This configuration represents a typical grid-connected microgrid architecture commonly investigated in energy management studies^3,5^, enabling flexible operation under varying conditions of renewable energy generation and load. Figure 1 illustrates the structure of the actual MG system.

The PV array and wind turbine generator act as intermittent RES, whose output power fluctuates according to environmental conditions such as solar irradiance and wind speed. These sources primarily supply the local load demand, while any surplus or deficit of energy is managed through the BESS and the utility grid. The BESS plays a key role in balancing generation and consumption by storing excess renewable energy and supplying power during periods of insufficient renewable generation.

The MG is assumed to operate in both grid-connected and islanded modes^12^. In grid-connected mode, the utility grid can import power to support the load when local generation and battery discharge are insufficient, or export excess power when renewable generation exceeds demand and battery charging limits are reached. In islanded operation, the grid contribution is unavailable, and the balance between generation and demand must be maintained solely through local renewable sources and the BESS.

The power balance within the MG is defined following the formulation used in the original control model. The net power balance is expressed as:

1 $${P}_{balance}= {P}_{load}-{P}_{DG}-{P}_{grid}$$

where ${P}_{\mathrm{load}}$ denotes the total load demand, ${P}_{\mathrm{DG}}$ represents the combined power generated by the renewable sources (PV and wind), and ${P}_{\mathrm{grid}}$ is the power exchanged with the utility grid. When the grid is disconnected, ${P}_{\mathrm{grid}}$ is set to zero. The renewable generation term is defined as:

2 $${P}_{DG}={P}_{PV}+{P}_{WT}$$

where ${P}_{\mathrm{PV}}$ and ${P}_{\mathrm{WT}}$ correspond to the photovoltaic and wind turbine power outputs, respectively.

Battery operation is limited by SOC bounds to ensure safe and reliable performance. In this study, the SOC is kept between a minimum of 20% and a maximum of 80%, aligning with common practices in microgrid energy management^3,12^. When the SOC hits its lower limit, discharging is blocked, and charging is activated when energy is available. Conversely, when the SOC reaches the upper limit, charging is stopped to prevent overcharging. These constraints are explicitly included in the control strategy described in the following sections.

### Phase 1—fuzzy logic control strategy

#### Fuzzy logic controller description

In the initial phase of this study, the FLC strategy proposed by Faisal et al.^12^ is adopted as the baseline control framework in order to enable a fair and consistent comparison with previously reported optimization approaches. To ensure comparability, the same MG configuration, operating conditions, and control variables are used. The main distinction lies in the tuning approach: while the original study employed PSO and the BSA, this work applies GA for fuzzy parameter optimization.

The FLC is designed with two input variables and one output variable. The first input is the power balance ${P}_{\mathrm{balance}}$, defined in Eq. (1), which represents the mismatch between load demand and available generation. The second input is the SOC deviation ΔSOC, defined as the difference between the actual battery SOC and a predefined reference value, expressed as:

3 $${\Delta} SOC={SOC}_{current}-{SOC}_{reference}$$

The output of the fuzzy controller is the battery current $I$, which determines the charging or discharging behavior of the BESS. Positive current values correspond to battery discharging, while negative values indicate charging.

Battery operation is constrained within predefined SOC limits to ensure safe and reliable performance. When the SOC reaches the lower threshold (20%), battery discharging is inhibited, and charging is enforced whenever energy is available. Conversely, when the SOC reaches the upper threshold (80%), battery charging is prevented to avoid overcharging. These constraints are incorporated directly into the control logic, following the same assumptions adopted in^3,12^.

Each fuzzy variable, ${P}_{\mathrm{balance}}$, ${\Delta} SOC$, and the output current $I$, is characterized using five linguistic MFs: Very Small (VS), Medium Small (MS), Normal (N), Medium Large (ML), and Very Large (VL). Based on these linguistic variables, a total of 25 fuzzy inference rules are defined, corresponding to all possible combinations of the two inputs. The fuzzy rule base, adopted from^12^, is summarized in Table 3.

**Table 3:** Fuzzy rules of the phase 1 controller.

Decision	*P*_*balance*_				
	VS	MS	N	ML	VL
**ΔSOC**					
VS	VS	VS	VS	VS	VS
MS	VS	VS	N	ML	ML
N	VS	MS	N	ML	ML
ML	VS	MS	N	ML	VL
VL	N	N	N	ML	VL

The rule base reflects intuitive battery management behavior. For example, when the power balance indicates a deficit and the battery SOC is sufficiently high, the controller commands battery discharging to support the load. Conversely, when renewable generation exceeds demand, and the battery SOC is low, charging is prioritized. These rules enable the controller to dynamically adapt battery operation in response to changing generation and load conditions.

#### Fuzzy logic tuning using genetic algorithm

To improve the performance of the FLC, the membership functions of both the input and output variables are optimized using GA. The objective of the tuning process is to enhance battery current regulation and SOC management, thereby reducing the risk of overcharging or deep discharging.

**Fig. 2:**
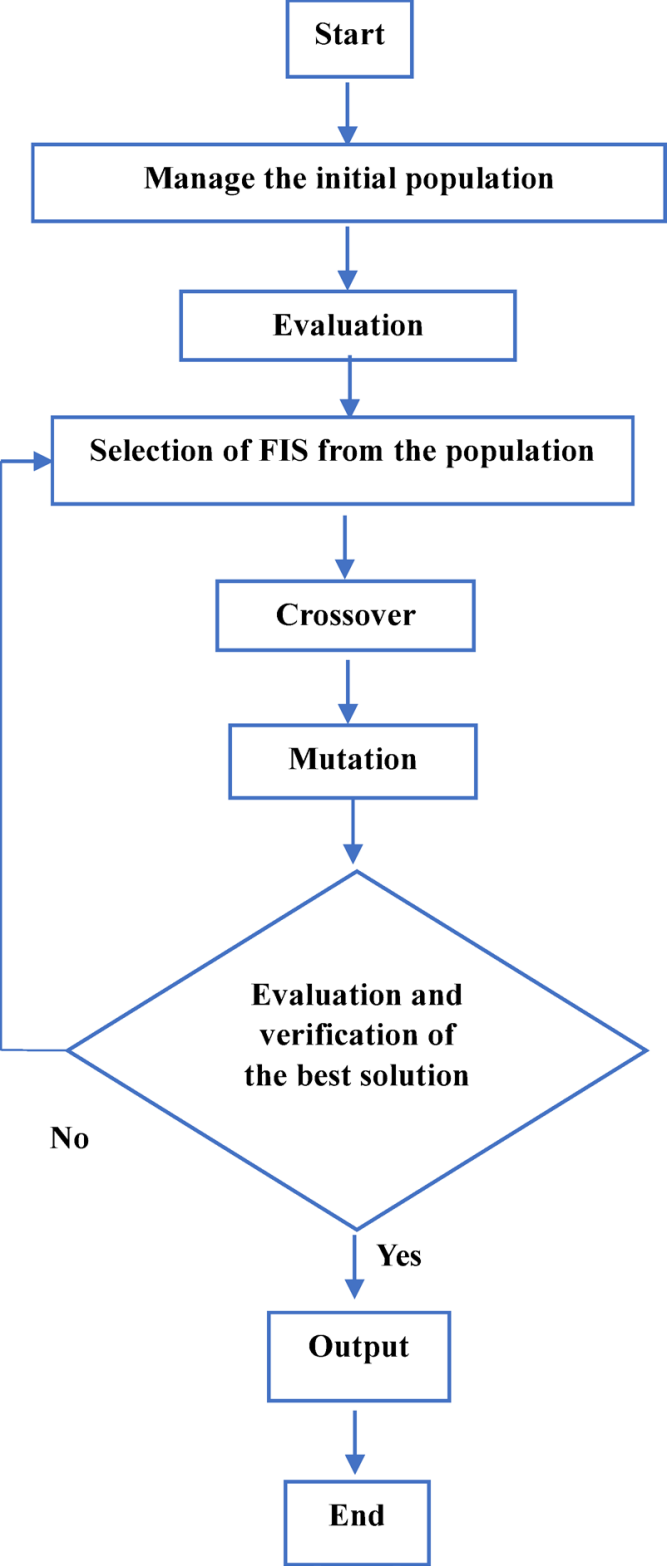
FIS-GA flowchart model.

The GA optimization process follows the flow illustrated in Fig. 2. An initial population of candidate solutions, each representing a specific set of fuzzy MF parameters, is generated randomly. Each candidate solution is evaluated using an objective function defined in accordance with^12^. The instantaneous current error is expressed as^12^:

4 $${I(t}_{i})= {({I}_{estimated}- {I}_{actual})}^{2}/N$$

where $N$ denotes the total number of time steps, ${I}_{\mathrm{estimated}}$ is the battery current estimated by the fuzzy controller, and ${I}_{\mathrm{actual}}$ is the actual battery current.

The overall objective function (*J*) is then formulated to minimize the cumulative error between the estimated and actual battery current^12^.

5 $$J= min\sum_{i=1}^{N}I({t}_{i})$$

Through successive generations, the GA applies selection, crossover, and mutation operators to evolve the population toward improved solutions. The selection process favors individuals with lower objective function values, while crossover and mutation introduce diversity and prevent premature convergence. The optimization procedure terminates when a predefined stopping criterion is satisfied, either when the maximum number of generations is reached or when the objective function converges.

The optimized fuzzy parameters obtained through this process are then applied to the controller and validated through time-domain simulations under the same operating conditions. The main GA parameters used in this study are listed in Table 4.

**Table 4:** FIS-GA tuning parameters.

Parameter	Estimated value
Population size	100
Crossover type	Arithmetic
Crossover probability	0.8
Mutation method	Gaussian
Mutation rate	0.01

### Phase 2—alternative control strategy with enhanced parameters

In this second phase, the control objective is extended beyond continuous current-based battery regulation (Phase 1) to an operational energy management strategy that explicitly coordinates renewable generation, battery operation, and grid interaction. While Phase 1 regulates the battery current to indirectly maintain balance, practical microgrid operation often requires explicit decisions regarding battery charge/discharge and grid import/export under SOC and power availability constraints. To address this, Phase 2 introduces an enhanced fuzzy inference system (FIS) that embeds these operational decisions within the controller.

#### Enhanced EMS logic and fuzzy controller structure

The enhanced strategy prioritizes direct use of RES and employs the battery energy storage system to manage power surpluses and deficits, while enforcing practical grid-interaction rules. Specifically:

- The battery charges when RES generation exceeds load demand, and SOC is below its upper limit.
- The battery discharges when RES generation is insufficient, and SOC is sufficiently high.
- Export to the grid is allowed only when generation exceeds demand, and the battery is at (or near) its maximum SOC.
- Import from the grid is allowed only when load demand exceeds RES generation, and the battery cannot compensate for the deficit due to SOC limitations.

**Fig. 3:**
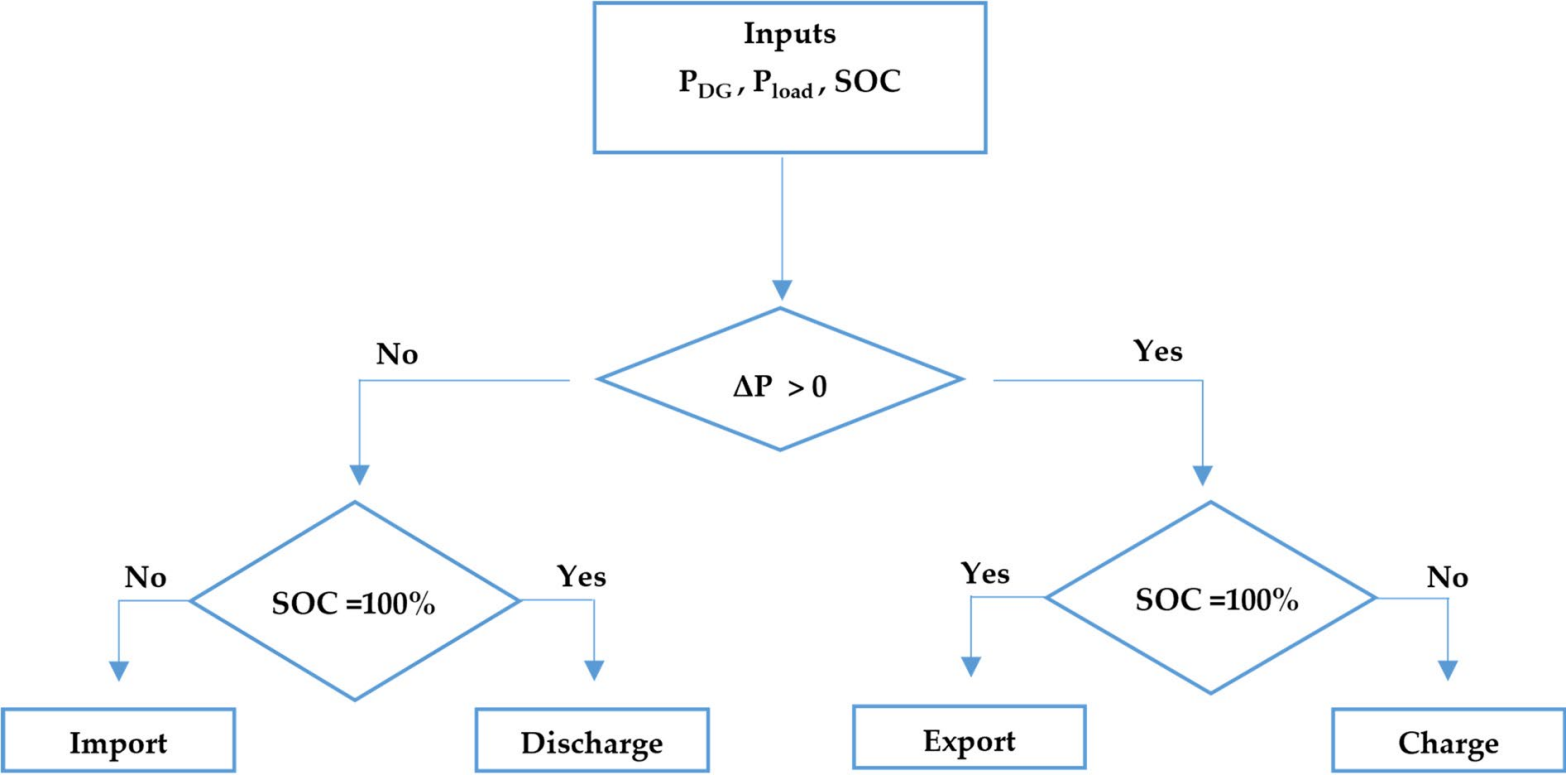
EMS control flowchart.

This rule-based logic is illustrated in Fig. 3, which summarizes the energy management system (EMS) flow and the logical conditions governing charge/discharge and import/export actions.

Compared with Phase 1, the enhanced fuzzy controller adopts two input variables: (i) the battery SOC and (ii) the power difference between RES generation and load demand, defined as:

6 $${\Delta }P={P}_{DG}-{P}_{load}$$

A positive $\Delta P$ indicates surplus renewable generation, whereas a negative $\Delta \mathrm{P}$ indicates an energy deficit that must be covered by battery discharge and/or grid import. Each input is represented using five linguistic MFs: Very Small (VS), Medium Small (MS), Normal (N), Medium Large (ML), and Very Large (VL), consistent with the linguistic structure of Phase 1^12^.

**Fig. 4:**
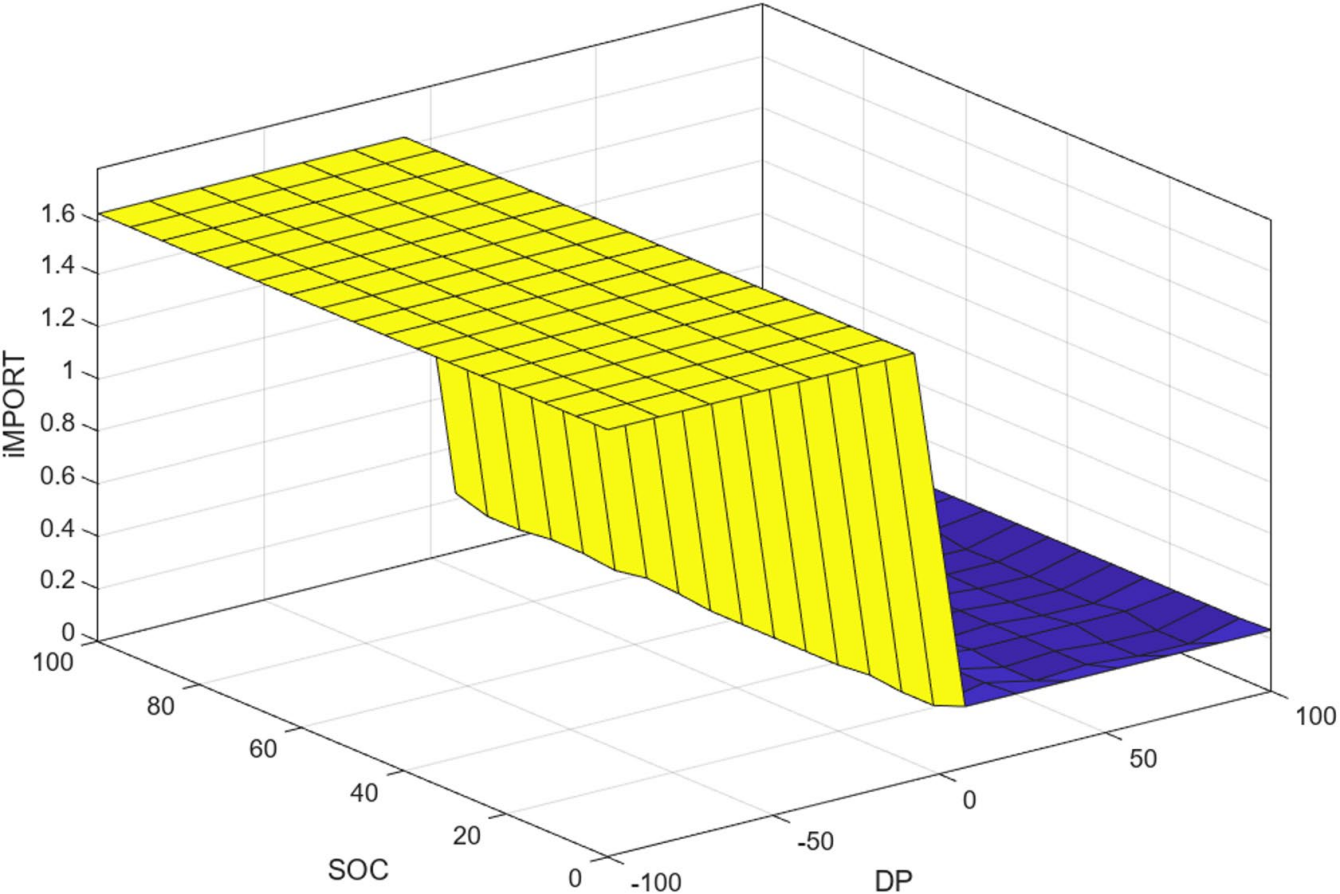
3D surface of the FIS as a function of the rules.

Unlike Phase 1, which produces a continuous current output, the Phase 2 controller generates discrete operational decisions: Charge, Discharge, Import, and Export. Each decision is represented by binary values (0: inactive, 1: active), resulting in a practical decision-driven EMS. The rule base defining these decisions is presented in Table 5, and the corresponding fuzzy control surface is visualized in Fig. 4.

**Table 5:** FL rules of the phase 2 controller.

Decision	∆P				
	VS	MS	N	ML	VL
**SOC**					
VS	VS	VS	VS	VS	VS
MS	VS	VS	N	ML	ML
N	VS	MS	N	ML	ML
ML	VS	MS	N	ML	VL
VL	N	N	N	ML	VL

#### Rule base and GA-based tuning

As in Phase 1, GA is employed to optimize the membership functions of the Phase 2 fuzzy controller to improve decision consistency and overall performance of the EMS. The GA-based fuzzy tuning process follows the same optimization rationale adopted in Phase 1 and is consistent with established GA-FLC tuning methodologies reported in the literature^12^.

The Phase 2 fuzzy inference system is constructed using a rule base comprising 25 rules, derived from all possible combinations of the linguistic variables associated with the battery SOC and the power difference ΔP, as summarized in Table 5. The rule base is designed to ensure balanced energy flows within the MG by prioritizing RES use, protecting battery operational limits, and minimizing unnecessary dependence on the utility grid. Several representative rules include:

- **ΔP = VS**, **SOC = MS** → *Import* = *1; Export* = *0; Charge* = *0; Discharge* = *0*: This scenario reflects a minor energy shortfall (ΔP < 0) with moderately low battery SOC. The system opts to import energy from the grid, as neither production nor stored energy can meet the demand.
- **ΔP = VL**, **SOC = VS** → *Import* = *0; Export* = *0; Charge* = *1; Discharge* = *0*: A significant energy surplus and a nearly depleted battery prompt the system to charge the battery, maximizing local RES usage and enhancing system autonomy.
- **ΔP = N**, **SOC = N** → *Import* = *0; Export* = *0; Charge* = *0; Discharge* = *0*: With energy supply and demand in balance and SOC at mid-level, no control action is required, indicating stable operation.
- **ΔP = VS**, **SOC = VL** → *Import* = *0; Export* = *0; Charge* = *0; Discharge* = *1*: A small energy deficit and high SOC prompt the battery to discharge, covering the load and avoiding unnecessary grid import.
- **ΔP = ML**, **SOC = VL** → *Import* = *0; Export* = *0; Charge* = *0; Discharge* = *1*: With high SOC and a moderate surplus, the system prioritizes battery discharge to prevent overcharging and maintain energy balance.

This enhanced rule-based strategy reflects a more realistic and operationally grounded EMS, in which control decisions explicitly coordinate battery operation and grid interaction rather than relying solely on continuous power regulation. By embedding import and export logic directly within the fuzzy inference system, the proposed approach aligns more closely with practical grid interconnection requirements and real-world MG operation.

## Results

### Phase 1—performance of the GA-tuned fuzzy controller

The performance of the GA-tuned fuzzy logic controller is evaluated under the same system configuration, operating conditions, and load-generation profiles as those reported in^12^, allowing for a direct and fair comparison with PSO and BSA-based tuning approaches. The analysis focuses on three key aspects: (i) optimization convergence behavior, (ii) battery current regulation, and (iii) SOC evolution over a 24-h operating cycle.

**Fig. 5:**
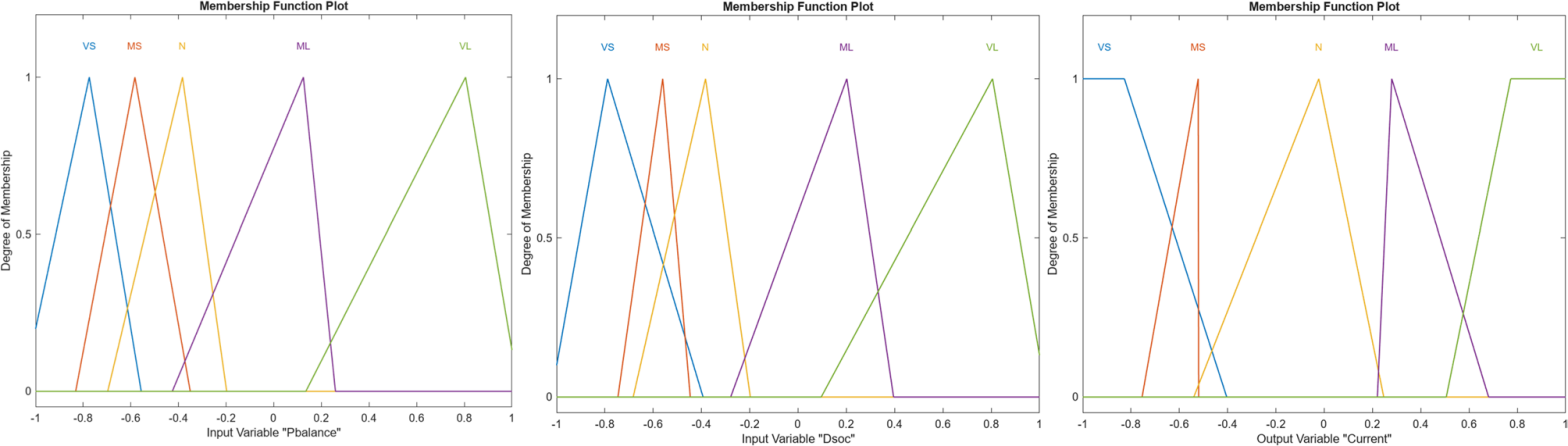
Tuned MFs of the fuzzy logic controller FIS-GA.

Figure 5 illustrates the MFs obtained after GA-based tuning. Compared to the initial fuzzy configuration, the optimized membership functions exhibit sharper transitions and reduced overlap between adjacent linguistic terms. This refinement enhances the controller’s sensitivity to power balance variations and SOC deviations, enabling more precise charging and discharging decisions. The optimized shapes indicate that the GA effectively adapts the fuzzy parameters to the nonlinear characteristics of the microgrid system.

**Fig. 6:**
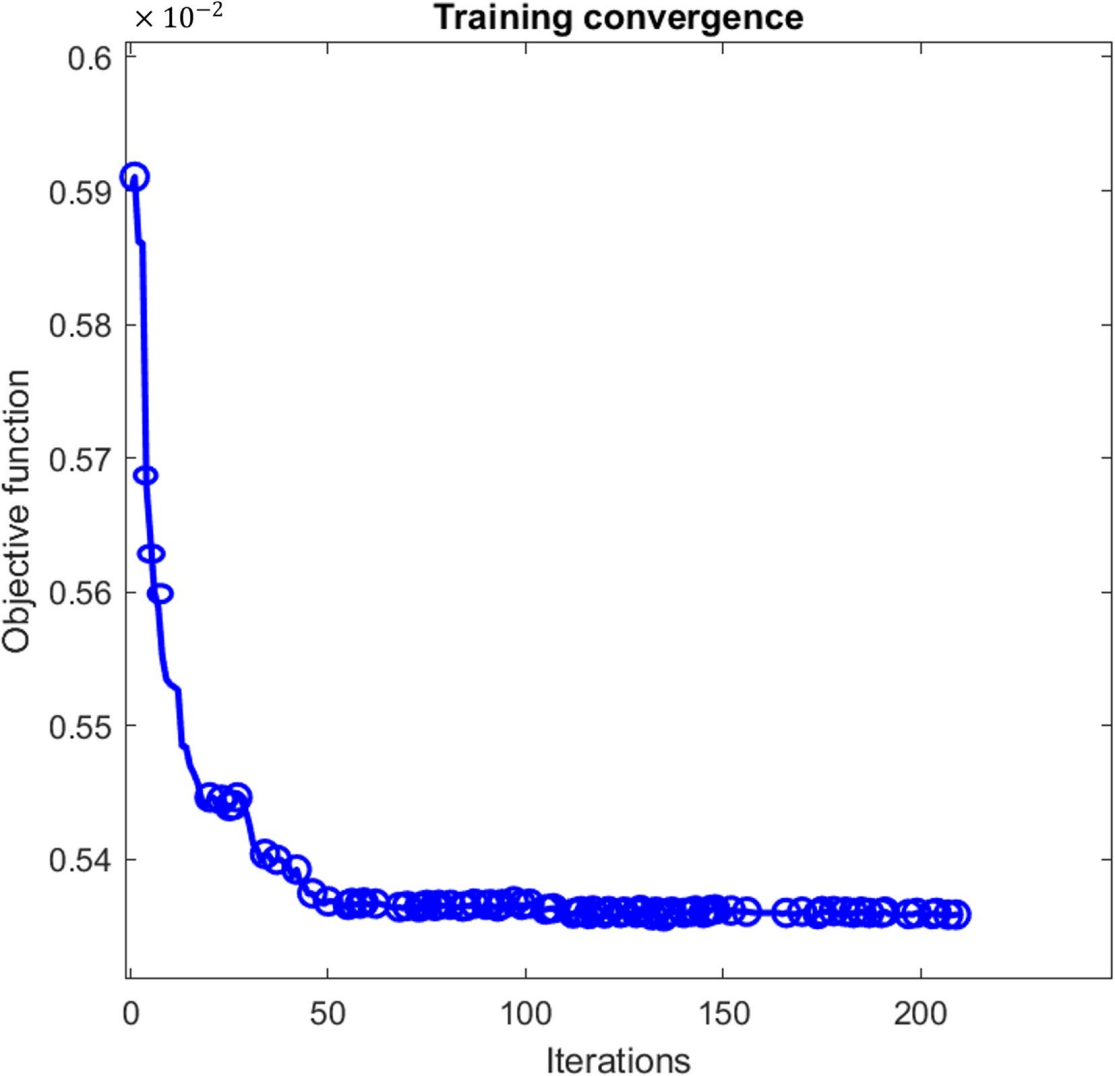
The convergence curves of the objective function for GA.

The convergence behavior of the GA optimization process is illustrated in Fig. 6 and compared with the convergence curves of PSO and BSA reported in^12^ (see Figures A.1 and A.2 in Appendix A). The GA reaches convergence after approximately 201 iterations, whereas BSA and PSO converge within 42 and 82 iterations, respectively. Although GA exhibits slower convergence, this behavior reflects its stronger emphasis on maintaining population diversity and exploring the global landscape. By maintaining genetic variation over successive generations, GA reduces the risk of premature convergence, which is particularly relevant in nonlinear and multimodal optimization landscapes, such as those encountered in the tuning of fuzzy controllers.

**Fig. 7:**
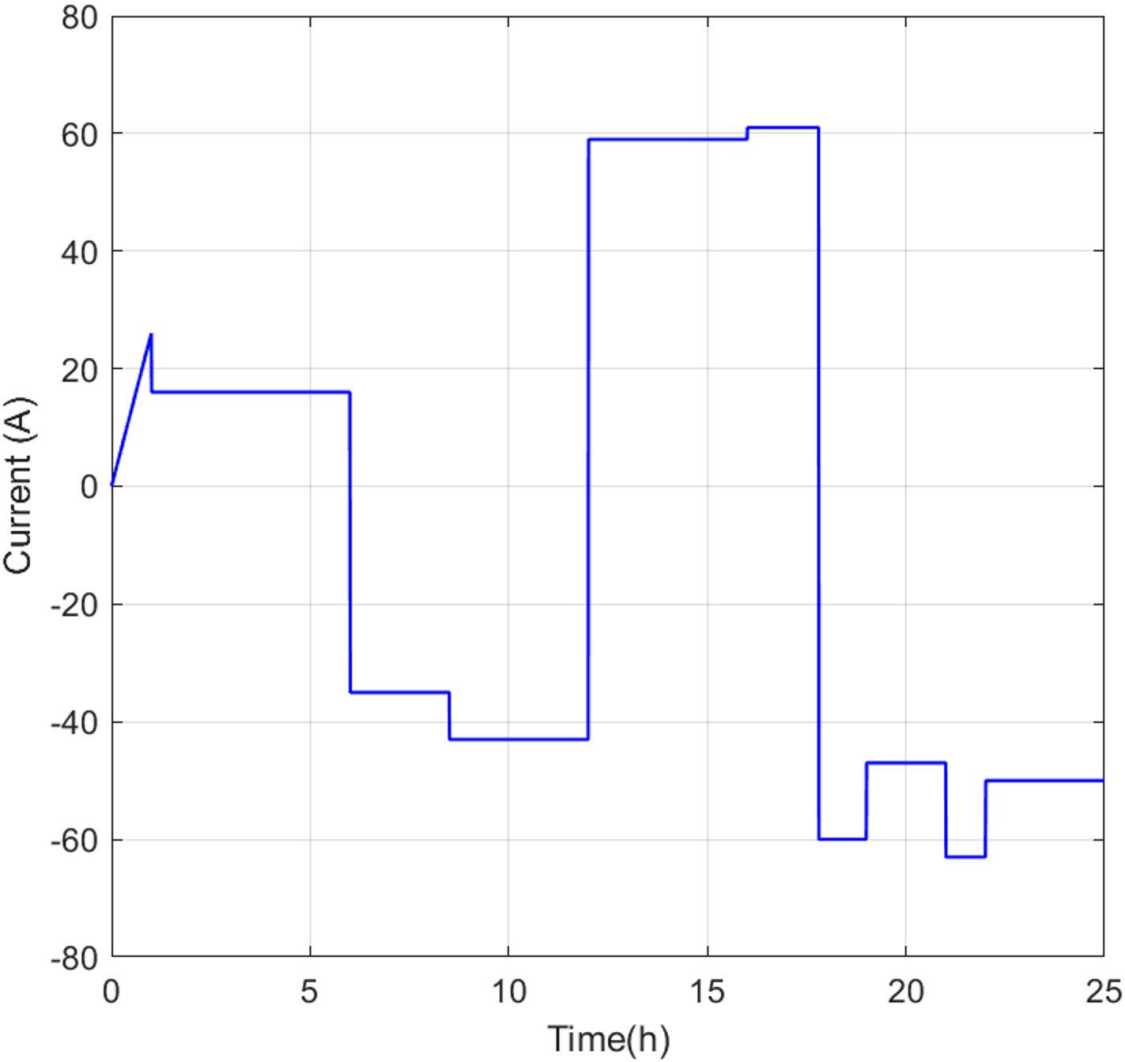
Battery output current for the FIS-GA.

Figure 7 shows the battery current output profile under FIS-GA control. Compared with FIS-BSA and FIS-PSO (see Figure A.3 in Appendix A), differences in the current trajectories are noticeable. For example, at 10:00, FIS-GA produces a current of -43 A, compared to -41 A with FIS-BSA. Similarly, at 08:00, FIS-GA delivers -35 A, while BSA yields -40 A. These differences, typically between 3 and 5 A, are minor and unlikely to significantly impact system performance during normal operation. However, they could become more important in long-term simulations or during stress testing, requiring future sensitivity analyses.

Operationally, when the grid is disconnected (00:00–06:00), the battery runs in discharge mode because distributed generation can’t meet the demand. From 06:00 to 12:00, renewables produce more than needed, so the battery charges. Between 12:00 and 16:00, demand increases again, and the battery discharges. During grid reconnection from 17:00 to 22:00, the system uses grid power while also charging the battery with any excess energy. Finally, between 22:00 and 24:00, even though the grid is off, renewable energy exceeds demand, and the battery continues to charge.

**Fig. 8:**
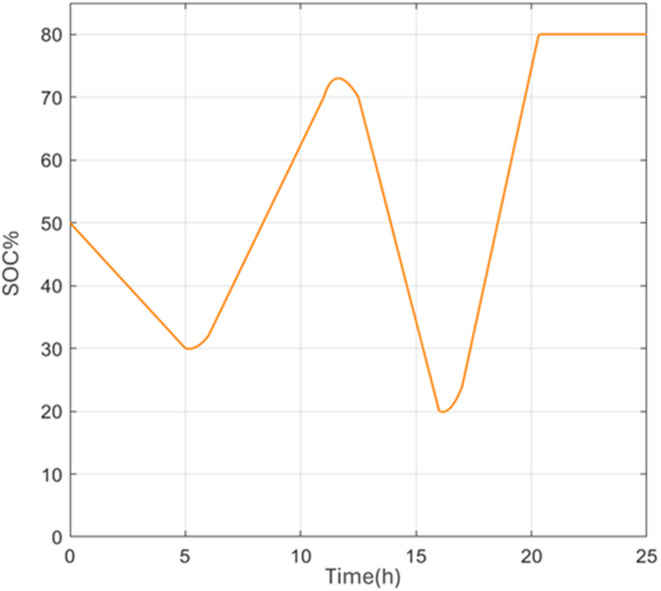
Battery state of charge with the GA model.

Figure 8 shows the SOC evolution corresponding to the GA-tuned controller, along with comparisons to PSO and BSA results (see Figure A.4 in Appendix A). All simulations start from an initial SOC of 50% and remain within the predefined safety limits of 20–80%. During the early discharge period, slight differences in SOC are observed among the methods; for instance, at 04:00, the SOC reaches approximately 34% for GA, compared to 36% for PSO and 40% for BSA. These deviations reflect differences in the aggressiveness of the discharge control rather than instability or constraint violation. As the simulation progresses, the SOC trajectories gradually converge, with all methods reaching similar final SOC values close to the upper limit by the end of the 24-h cycle.

Overall, the Phase 1 results confirm that the GA-tuned fuzzy controller achieves control performance comparable to that of PSO- and BSA-based approaches for battery-output current regulation and SOC management. While BSA exhibits faster convergence and slightly lower objective function values, the GA provides stable and robust control behavior without violating operational constraints. These results validate the use of GA as a viable tuning method and establish a consistent baseline for evaluating the enhanced control architecture introduced in Phase 2.

### Performance of the enhanced FIS-GA controller

Phase 2 evaluates the performance of the enhanced FL–based energy management strategy introduced in Section "Phase 2 – Alternative Control Strategy with Enhanced Parameters". Unlike Phase 1, which focuses on continuous battery current regulation, this phase emphasizes decision-based coordination among renewable generation, battery operation, and grid interaction. The assessment, therefore, focuses on the controller’s ability to manage SOC, regulate grid import/export behavior, and maintain stable electrical performance under variable operating conditions.

**Fig. 9:**
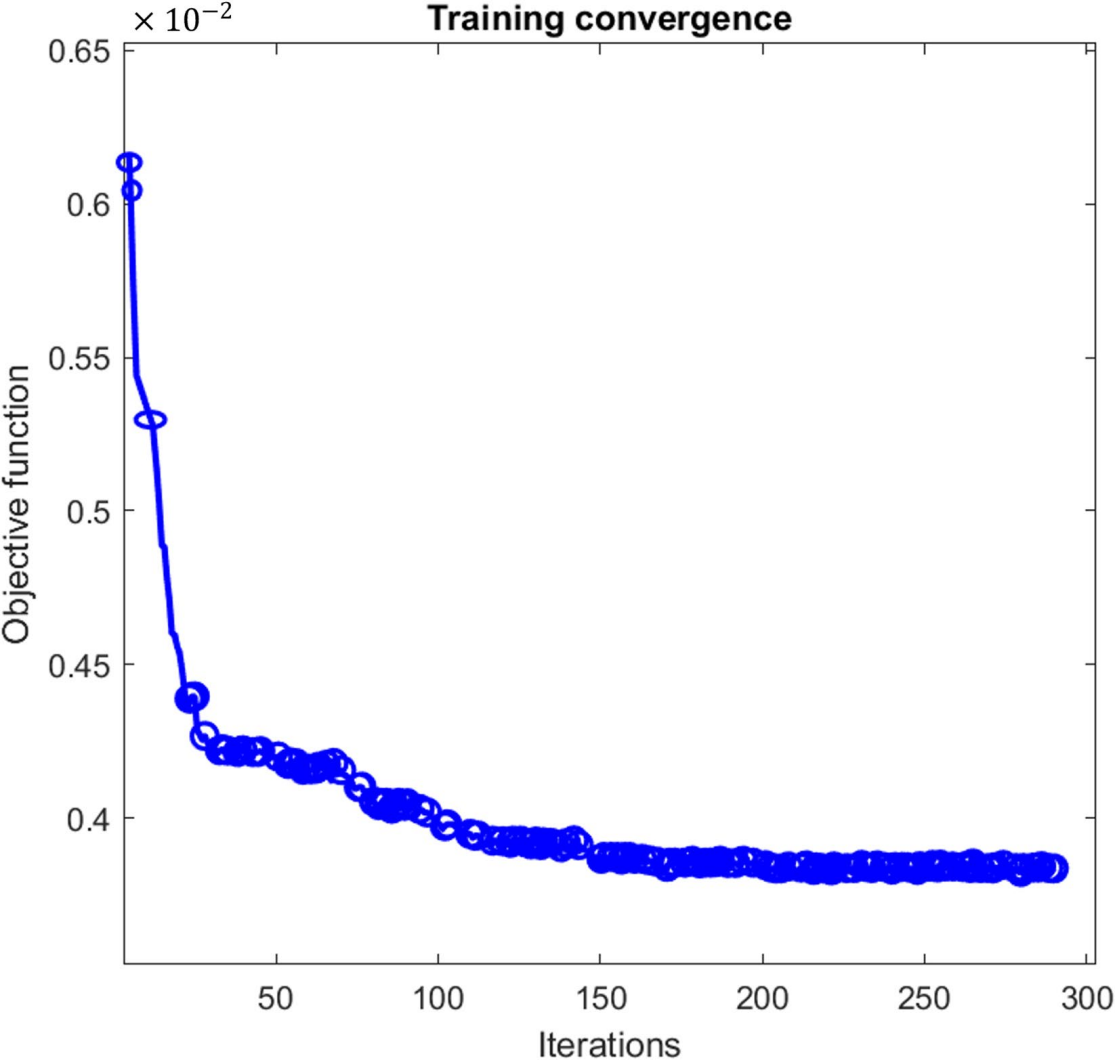
Optimization convergence curve for the modified FIS-GA.

Figure 9 illustrates the convergence behavior of the GA during the tuning of the enhanced fuzzy controller. Compared to Phase 1, the optimization process requires a significantly larger number of iterations (approximately 290) to reach convergence. This increase is expected and reflects the higher dimensionality of the optimization problem, as the controller now handles multiple discrete decisions (charging, discharging, import, and export) instead of a single continuous output. Despite the increased complexity, the GA converges smoothly without oscillations, indicating stable optimization behavior.

**Fig. 10:**
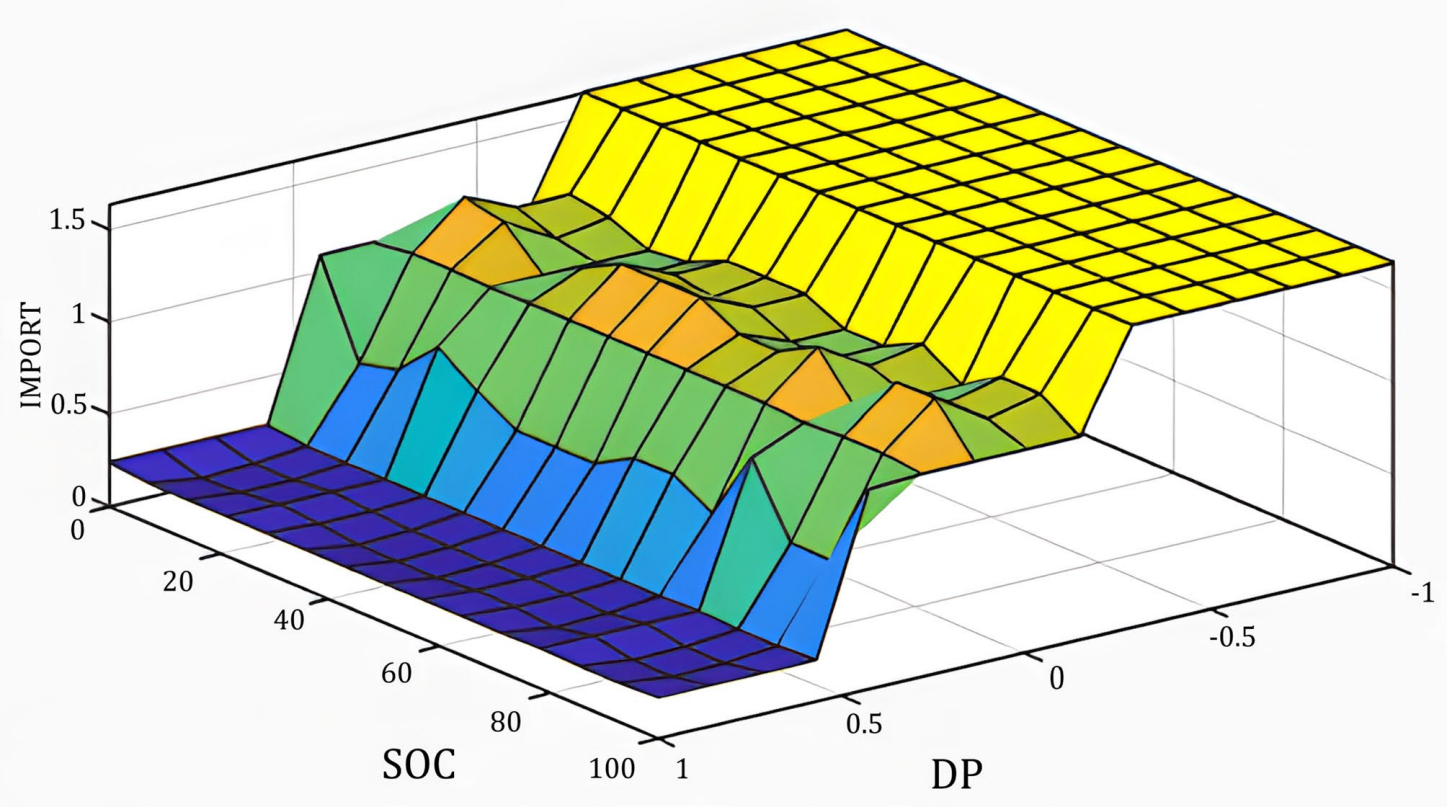
3D surface of the rule-based optimized system.

The optimized fuzzy control surface shown in Fig. 10 highlights the nonlinear decision boundaries generated by the rule-based energy management system. The surface demonstrates how variations in power difference (ΔP) and SOC jointly influence control decisions. Smooth transitions between operating regions indicate that the controller avoids abrupt switching behavior, which is desirable for the practical operation of a microgrid.

**Fig. 11:**
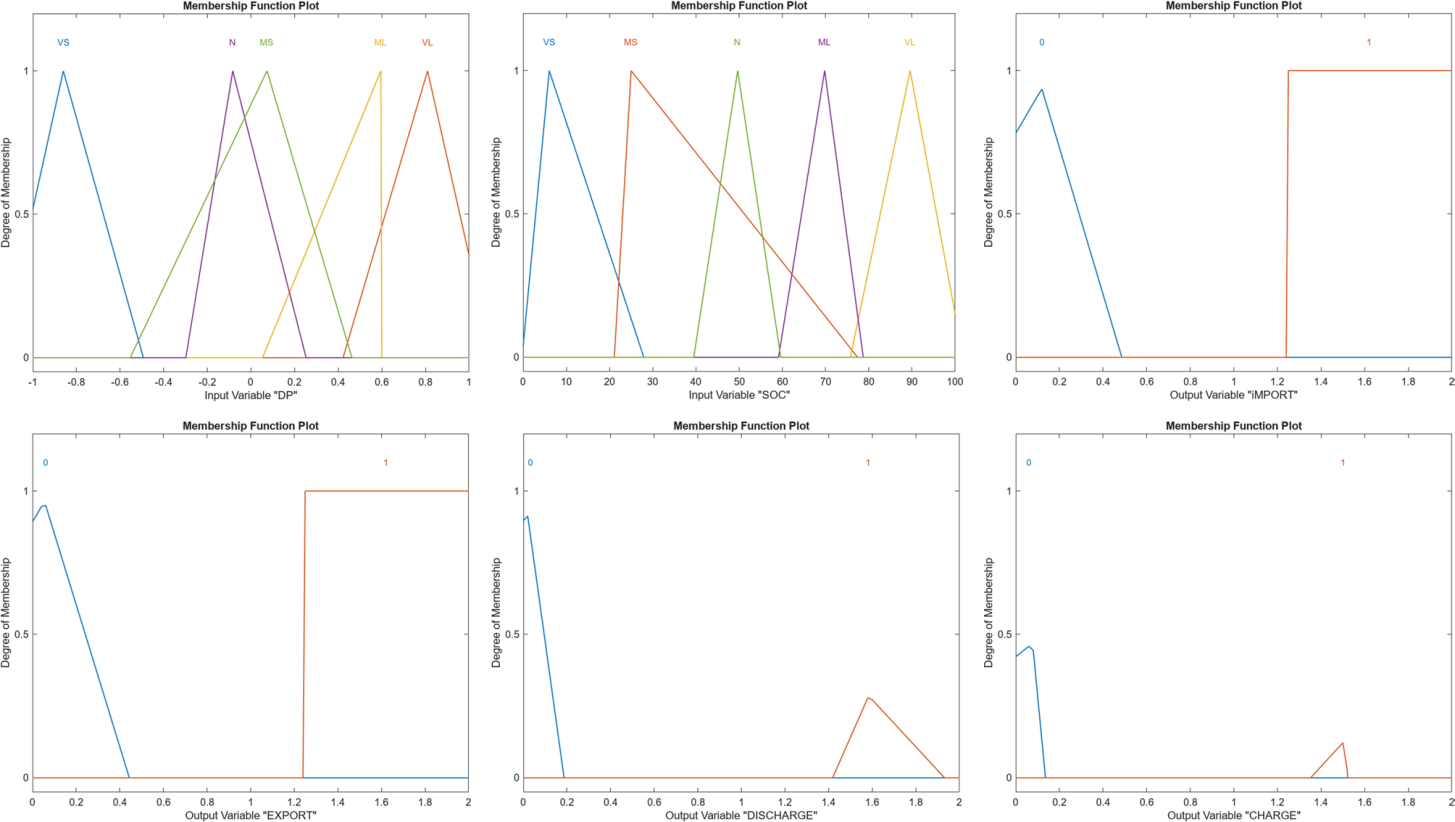
The optimized inputs and outputs MFs.

Figure 11 presents the tuned MFs of the enhanced fuzzy controller. The overall effect of this tuning process is to enable a more adaptive and nuanced control strategy, one that aligns with real-world fluctuations in demand and RES generation while adhering to logical export/import rules. Compared to the Phase 1 configuration, greater overlap is observed between adjacent linguistic terms, particularly at intermediate SOC and ΔP values. This overlap supports gradual transitions between control actions and enhances decision robustness under borderline operating conditions, such as near-zero power imbalance or mid-range SOC levels.

**Fig. 12:**
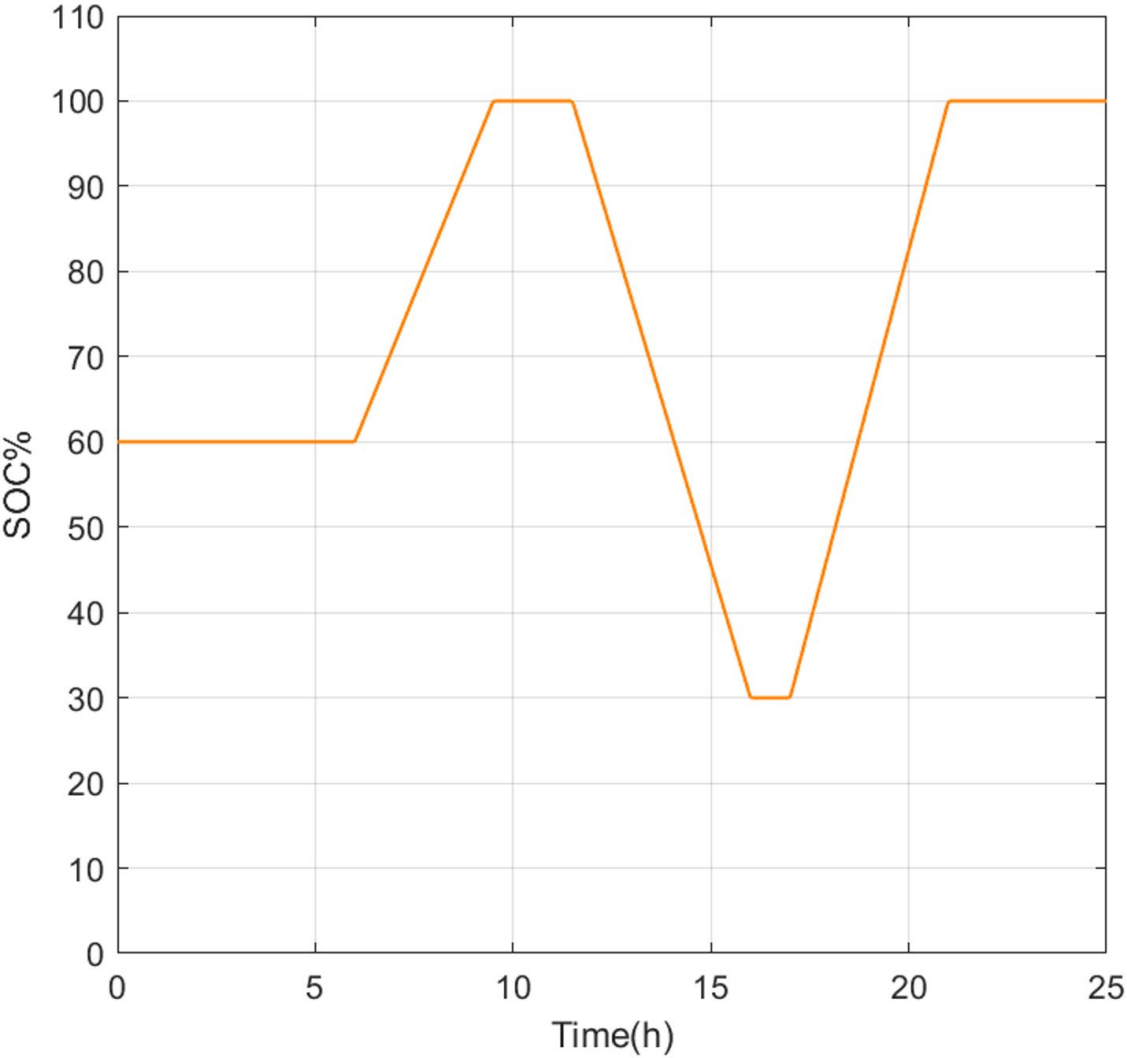
The SOC variation of the modified FIS-GA.

The SOC trajectory resulting from the enhanced FIS-GA controller is shown in Fig. 12. The SOC evolution exhibits a clear operational logic consistent with the EMS rules described in Section "Phase 2—alternative control strategy with enhanced parameters". During periods of insufficient renewable generation, such as early morning hours and around 16:00, grid import is activated to support the load while preventing excessive battery discharge. Conversely, during periods of surplus renewable generation, the battery is charged preferentially until it reaches its upper SOC limit. Export to the grid occurs only when the battery is fully charged, and renewable generation continues to exceed demand.

Compared to Phase 1 results, the SOC profile in Phase 2 shows longer periods of high SOC stability, indicating more effective use of available renewable energy and reduced cycling stress on the battery. Several intervals show the SOC remaining at or near its maximum value, reflecting either balanced generation and demand conditions or surplus energy that cannot be further stored or exported due to system constraints. This behavior highlights the controller’s ability to enforce operational limits while maintaining system stability.

**Fig. 13:**
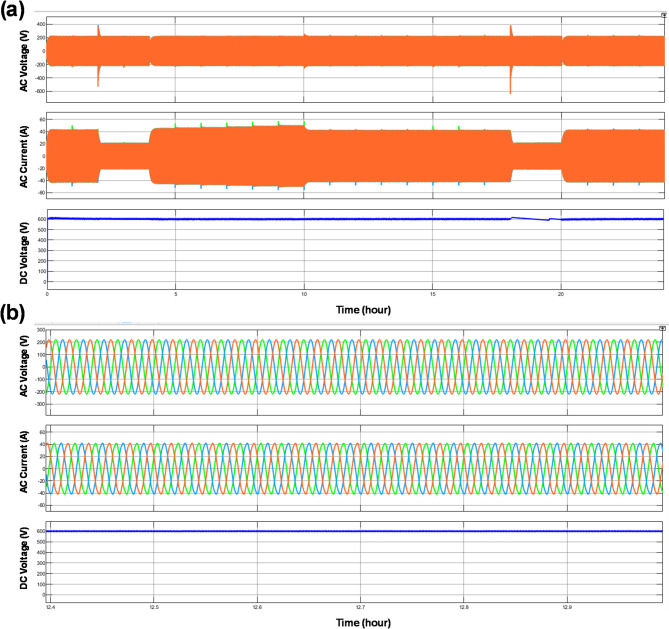
The variation in voltage, current, and forward voltage of the system.

Electrical performance under the enhanced control strategy is evaluated using the voltage and current waveforms shown in Fig. 13. Figure 13a demonstrates that the three-phase voltages and currents remain balanced and well synchronized throughout the operating period, despite frequent changes in control actions. Minimal distortion and stable amplitude indicate effective coordination between the EMS decisions and the underlying power electronic interfaces. Figure 13b further confirms symmetrical waveform behavior, suggesting that the enhanced decision-based controller does not introduce adverse electrical transients.

Overall, the Phase 2 results demonstrate that embedding grid interaction logic directly within the fuzzy inference system significantly improves the practicality and coherence of microgrid energy management. While the enhanced controller requires greater computational effort during the optimization stage, it delivers more realistic operational behavior, improved SOC regulation, and stable electrical performance. These results confirm that architectural enhancements of the fuzzy controller yield greater performance benefits than tuning alone, particularly in grid-connected microgrid applications.

## Discussions

The results presented in Sections "Phase 1—performance of the GA-tuned fuzzy controller" and "Performance of the enhanced FIS-GA controller" highlight two complementary aspects of FL-based energy management in microgrids: the influence of optimization algorithms on controller tuning and the impact of controller architecture on overall system performance.

The Phase 1 analysis demonstrates that different population-based metaheuristic algorithms, namely GA, PSO, and BSA, yield comparable system-level performance once convergence is achieved. Although BSA converges faster and attains slightly lower objective function values, all three approaches maintain battery SOC within safe limits and produce similar current and SOC trajectories. This observation is consistent with previous studies, which report that, for a fixed fuzzy controller structure, the choice of optimization algorithm primarily affects convergence behavior rather than long-term operational performance^3,15,16,30^. These findings suggest that further improvements in MG energy management cannot rely solely on increasingly sophisticated tuning algorithms.

In contrast, the architectural enhancement introduced in Phase 2 leads to more substantial and practically relevant performance improvements. By embedding battery charge–discharge decisions and grid import–export logic directly within the fuzzy inference system, the enhanced controller achieves more coherent coordination between renewable generation, storage, and grid interaction. The resulting SOC profiles exhibit longer periods of stability near upper limits, indicating improved utilization of surplus renewable energy and reduced unnecessary battery cycling.

Similar trends have been observed in recent rule-based and hierarchical energy management approaches. For instance, the works by Nayak et al.^24^ and Prusty et al.^27^ share a common objective with the present study, namely, improving MG energy management through intelligent, optimized FLC. In these studies, fuzzy controllers are employed to regulate power balance and battery SOC under renewable intermittency, with controller parameters tuned using selected metaheuristic or hybrid optimization techniques. The reported results demonstrate the feasibility and effectiveness of the proposed control schemes under predefined operating scenarios. However, a key methodological distinction exists between these approaches and the present work. In the referenced studies, the fuzzy controller architecture is treated as fixed, and performance improvements are primarily attributed to the selected optimization algorithm. Consequently, the individual impact of the tuning strategy is not explicitly isolated from that of the controller structure. In contrast, the present study deliberately separates these two aspects by evaluating optimization effects on a fixed controller in Phase 1, followed by an explicit architectural enhancement in Phase 2. Moreover, several of the referenced approaches rely on multi-layer supervisory control or hybrid optimization frameworks, which can increase implementation complexity and reduce interpretability. The proposed strategy demonstrates that restructuring the fuzzy inference system to embed grid-interaction logic yields more substantial, operationally meaningful improvements in microgrid energy management, even when using standard population-based optimization methods.

A comparative summary of the tuning methods employed in Phase 1 is presented in Table 6, highlighting the trade-offs among convergence speed, robustness, and implementation complexity. While BSA shows advantages in convergence efficiency, the GA demonstrates greater flexibility when applied to more complex control architectures, as evidenced by its successful application in Phase 2. This scalability is particularly relevant for microgrids with evolving operational requirements.

**Table 6:** Comparison of GA-, PSO-, and BSA-based FLC tuning approaches (Phase 1).

Criterion	GA	PSO	BSA
Convergence speed	Moderate	Fast	Very fast
Objective function value	Comparable	Comparable	Slightly lower
Battery current regulation	Comparable	Comparable	Comparable
SOC constraint satisfaction	Yes	Yes	Yes
Sensitivity to initial population	Low	Moderate	Moderate
Scalability to complex controllers	High	Moderate	Moderate

Table 7 further illustrates the fundamental differences between the two phases of this study. The transition from continuous current-based regulation to a decision-based energy management strategy results in improved adaptability to renewable variability, explicit enforcement of grid interaction constraints, and more realistic operational behavior. These findings align with recent reports emphasizing the importance of controller structure and decision logic in achieving effective energy management in renewable-dominated MG^26^.

**Table 7:** Comparison between Phase 1 and Phase 2 fuzzy control strategies.

Aspect	Phase 1	Phase 2
Control objective	Continuous battery current regulation	Decision-based energy management
Controller inputs	Power balance, SOC deviation	Power difference (ΔP), SOC
Controller outputs	Battery current reference	Charge / Discharge / Import / Export
Grid interaction handling	External or implicit	Embedded within fuzzy inference
EMS decision granularity	Continuous	Discrete, rule-based
Battery utilization	Moderate	Improved (reduced unnecessary cycling)
Practical EMS realism	Limited	High
Architectural novelty	No	Yes

From a computational perspective, the enhanced controller incurs additional offline optimization cost due to its expanded decision space. However, this increase does not affect real-time implementation, as fuzzy inference remains computationally lightweight. The stable voltage and current waveforms observed under Phase 2 operation indicate that the proposed strategy maintains acceptable electrical performance despite frequent decision changes, suggesting adequate robustness under the tested conditions.

Nevertheless, several limitations should be acknowledged. The study assumes ideal measurement of SOC and power variables and does not explicitly model battery degradation or forecast uncertainties. Moreover, the analysis is restricted to a single MG configuration and a fixed set of operating scenarios. Future work should extend the proposed framework to account for battery aging, parameter uncertainty, and adaptive or predictive control elements.

## Conclusion

This paper investigated FL-based battery energy management strategies for a renewable-integrated MG, with particular emphasis on the combined effects of optimization techniques and controller architecture. A two-phase control framework was developed and evaluated using a hybrid MG comprising PV generation, wind energy, battery energy storage, and a bidirectional grid connection.

In Phase 1, GA was employed to tune an established FLC under identical operating conditions previously reported for PSO and BSA-based approaches. The results showed that, although the GA converged more slowly, all three metaheuristic methods achieved comparable system-level performance. Battery current deviations remained within approximately 3–5 A, and SOC variations differed by only a few percentage points, with all controllers maintaining SOC within the prescribed 20–80% safety limits. These findings confirm that, for a fixed fuzzy controller structure, optimization strategy primarily affects convergence behavior rather than long-term operational performance.

In Phase 2, the fuzzy controller architecture was enhanced by embedding battery charge–discharge decisions and grid import–export logic directly within the fuzzy inference system. This architectural modification led to more coherent and realistic energy management behavior. Simulation results demonstrated improved use of renewable energy, reduced unnecessary battery cycling, and clearer coordination between battery operation and grid interaction. Compared to Phase 1, the enhanced controller exhibited longer periods of SOC stability near upper limits and maintained stable, balanced voltage and current waveforms under dynamic operating conditions.

The key contribution of this study lies in demonstrating that controller architecture has a greater impact on the practical performance of MG energy management than the tuning strategy alone. While advanced optimization techniques remain important, structural enhancements to FLC can yield more substantial and operationally meaningful improvements without increasing real-time computational burden.

Despite these improvements, some limitations need to be acknowledged. The analysis assumes ideal measurement of system variables and does not explicitly account for battery degradation, forecasting uncertainty, or communication delays. In addition, the evaluation is limited to a single MG configuration and predefined operating scenarios.

The results of this study open several directions for future research. The observed trade-off between convergence speed and optimization accuracy suggests that hybrid or adaptive tuning strategies could further enhance fuzzy controller performance. In addition, the improved control behavior obtained with the redesigned fuzzy controller indicates that further refinement of the input–output structure, including the incorporation of predictive or uncertainty-aware variables, may improve decision-making under fluctuating renewable generation and load conditions. Finally, experimental validation or real-time implementation on hardware-in-the-loop platforms would further strengthen the applicability of the proposed approach to practical microgrid deployments.

## Supplementary Information

Below is the link to the electronic supplementary material.

Supplementary Material 1

## Data Availability

The data supporting the findings of this study are publicly available at Zenodo, under the accession number/DOI:10.5281/zenodo.15481709.
