# Supplementary material for: Comparative evaluation and architectural enhancement of a genetic algorithm-tuned fuzzy logic battery control in microgrid energy management
Source: Sci Rep. 2026 Mar 19;16:14209. doi: 10.1038/s41598-026-43620-7 (PMC13139369; doi:10.1038/s41598-026-43620-7)
Supplement: Supplementary file 1 — Supplementary Material 1 [file 41598_2026_43620_MOESM1_ESM.pdf]

Appendix A

Appendix A presents the reference figures from [12] illustrating the fuzzy tuning results for the MFS, the convergence curve, the current output, and the SOC using BSA and PSO algorithms. It serves as a benchmark for comparison with the outcomes of our own tuning simulation.

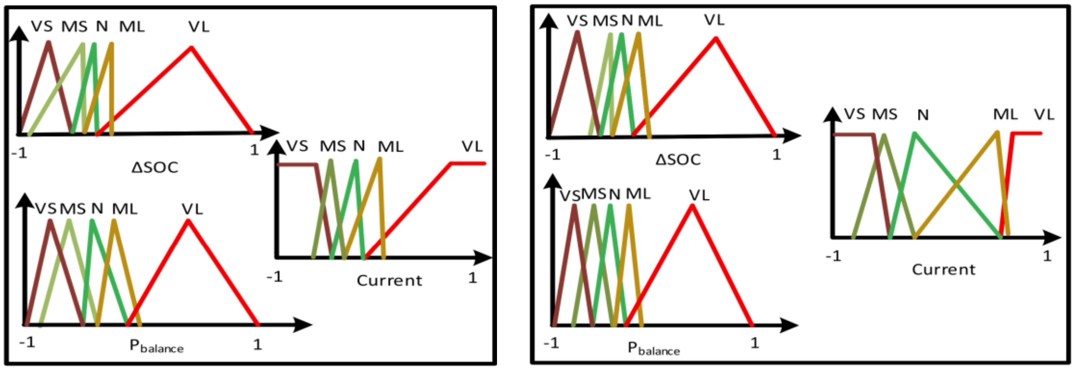

Figure A.1. MFs of the tuning FIS for (a) BSA, (b) PSO [12].

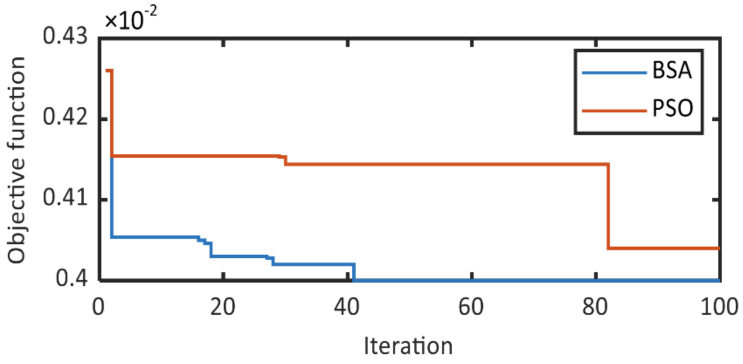

Figure A.2. The convergence curve of BSA and PSO methods [12].

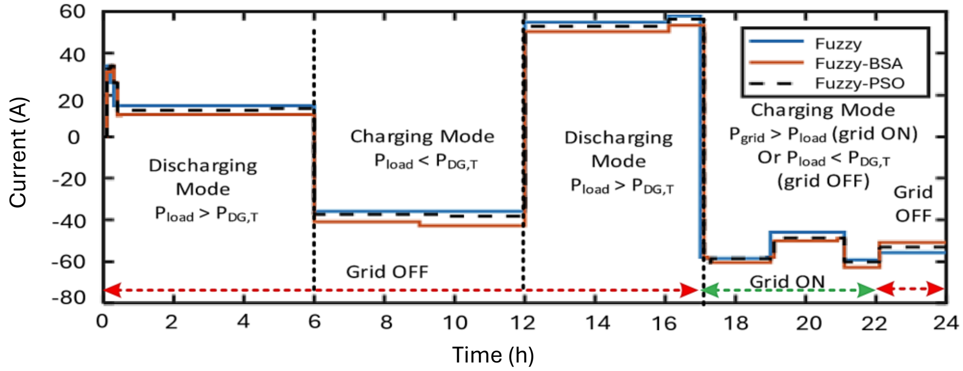

Figure A.3. Current output for fuzzy logic and optimized fuzzy and optimized fuzzy with both BSA and PSO [12].

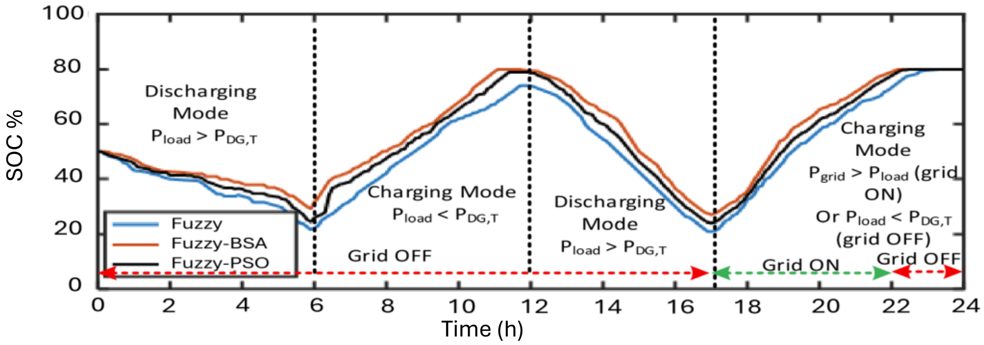

Figure A.4. SOC output with fuzzy and optimized both with fuzzy-BSA, and fuzzy-PSO [12].

References

[12] M. Faisal, M.A. Hannan, P.J. Ker, M.N. Uddin, Backtracking Search Algorithm Based Fuzzy Charging-Discharging Controller for Battery Storage System in Microgrid Applications, IEEE Access 7 (2019). https://doi.org/10.1109/ACCESS.2019.2951132.
